# Las relaciones personas-espacio público: Reflexiones sobre transformaciones, usos normativos, reducciones y contradicciones del espacio público en pandemia

**DOI:** 10.18294/sc.2023.4583

**Published:** 2023-12-22

**Authors:** Luz Adriana Muñoz-Duque, Nidia Elena Ortiz

**Affiliations:** 1 Doctora en Salud Pública. Docente, Facultad de Ciencias Sociales y Humanas, Facultad Nacional de Salud Pública, Universidad de Antioquia, Medellín, Colombia. luza.munoz@udea.edu.co Universidad de Antioquia Facultad de Ciencias Sociales y Humanas Facultad Nacional de Salud Pública Universidad de Antioquia Medellín Colombia luza.munoz@udea.edu.co; 2 Magíster en Psicología. Docente, Departamento de Psicología, Universidad de Antioquia, Medellín, Colombia. nidia.ortiz@udea.edu.co Universidad de Antioquia Departamento de Psicología Universidad de Antioquia Medellín Colombia nidia.ortiz@udea.edu.co

**Keywords:** COVID-19, Áreas Urbanas, Espacio Público, Inequidades, COVID-19, Urban Area, Public Space, Inequalities

## Abstract

Sobre la base del carácter espacial de los seres humanos y de la producción social de espacios y lugares, y considerando que la crisis sociosanitaria asociada al covid-19 derivó en cambios en y de los espacios, y en restricciones y mayores regulaciones de los usos espaciales y de las interacciones sociales, este artículo propone una reflexión teórica que aborda las restricciones pandémicas y la relación de las personas con el espacio público en el caso de Colombia. Tal reflexión es desarrollada en torno a cuatro vías de análisis: las transformaciones de la espacialidad pública en tanto materialidad; los usos normativos del espacio público como forma de vigilancia y control de los cuerpos; la reducción del mundo a escalas proximales del lugar y las consecuentes transformaciones en las relacionalidades con y en el espacio público; y, las contradicciones e inequidades del espacio público, expresadas en la distribución desigual de las formas espaciales de vivir la pandemia. Estos aspectos nos llevan a considerar la relevancia de rescatar el sentido vivencial de los espacios públicos, a partir del análisis de nuevas relacionalidades y redes sociales que allí tienen lugar..

## Abstract (en)

Based on the spatial aspect of human beings and the social production of spaces and places, and considering that the social and health crisis associated with COVID-19 led to changes in spaces as well as restrictions and greater regulations regarding their use and social interactions, this article develops a theoretical discussion on the relationship between people and public space in the context of Colombia. This reflection is developed around four themes: the transformations of public spatiality as materiality; the normative uses of public space as a way of surveillance and control of society; the lessening of the world to proximal spatial scales and the consequent transformations in the relationalities with and in public spaces; and the contradictions and inequities of public space, expressed in the unequal distribution of spatial forms of living the pandemic. These aspects lead us to consider the relevance of highlighting the experiential sense of public spaces, from the analysis of new relationalities and social networks that take place there.

## INTRODUCCIÓN

Los seres humanos somos intrínsecamente espaciales, en permanente compromiso con la actividad colectiva de producir espacios y lugares, y en esta particular espacialidad estamos implicados en complejas relaciones con nuestros entornos. Esta experiencia como seres espaciales tiene lugar en distintos niveles o escalas, que van desde el cuerpo, hasta geografías próximas (cuartos, casas, barrios) y otras más distantes (ciudades, regiones, países, mundo global). En todo caso, cada escala de esta espacialidad es producto de la acción e intención humanas colectivas y, por lo tanto, es susceptible de transformación, tanto activa e intencionada, como a partir de tensiones, conflictividades, opresiones, aperturas, lo político, lo ideológico, el conocimiento, el poder^1^. A propósito, plantea Kuri:

El espacio es ese lienzo de variada escala -desde los lugares donde se efectúan relaciones cara a cara, hasta los grandes conflictos geopolíticos- en donde se imbrican y cristalizan la historicidad, el poder, la cultura, la dominación y la resistencia, la identidad, la subjetividad y la memoria; en síntesis, la experiencia humana que, como se puede inferir, es una experiencia espacializada.^2^

Desde esta lógica, el espacio es una construcción social^3^; en él se inscriben las marcas de las dinámicas del poder, de la cultura y del devenir histórico. Su doble faceta, material y simbólica, está vinculada con la forma en que los sujetos, en constante interacción, se apropian de él. Cada persona y cada sociedad cuentan con una forma específica de concebir, apropiarse, relacionarse, organizar y nombrar el espacio y el tiempo y, al hacerlo, se configuran poco a poco a sí mismas; así, se constituyen espacios de trabajo y de descanso, de ocio y de castigo, profanos y sagrados, de celebración y de memoria. Consecuentemente, las prácticas definen los usos sociales del espacio, mientras los espacios institucionalizados hacen posible y constriñen ciertos tipos de prácticas y relaciones sociales^2^. 

Siguiendo a Moser^4^, las relaciones entre las personas y los lugares tienen asiento en distintos niveles de referencia espacial: el microambiente, que alude al espacio privado; los ambientes de proximidad, que son los espacios compartidos o semipúblicos; los ambientes públicos (ciudad, campo, entre otros), y el ambiente global. Esta lógica de escalas espaciales comprende no solo un ambiente físico (dimensión material), sino también un aspecto social, en la medida en que con cada nivel se involucran más personas y las relaciones son más distantes; igualmente, cuando el nivel es mayor, menor es la posibilidad de control y dominio del ambiente. 

Este trabajo centra la reflexión en el nivel de referencia espacial de los ambientes públicos, particularmente, en el espacio público como parte constitutiva de las ciudades. Cabe señalar que, en la Carta Mundial por el Derecho a la Ciudad^5^, la ciudad es definida como un espacio colectivo que pertenece a todos sus habitantes y es culturalmente rico y diverso. Desde esta perspectiva, el concepto de ciudad presenta dos vías de comprensión: en la primera, por su dimensión física, la ciudad es una metrópoli, urbe, villa o poblado que se encuentra organizada institucionalmente como una unidad local de gobierno de carácter municipal o metropolitano e incluye los espacios urbanos, rurales o semirrurales. La segunda acepción comprende la ciudad como espacio político, como un conjunto de instituciones y actores: gubernamentales, los cuerpos legislativo y judicial, movimientos y organizaciones sociales y, en general, la comunidad^5^. En la ciudad se llevan a cabo procesos interactivos, de regulación y comunicativos, los cuales se constituyen en la base sobre la que se sostiene el resto de los elementos que la conforman. Los componentes de la ciudad se articulan entre ellos; en consecuencia, cuando las personas e instituciones se relacionan entre sí en el ámbito de la ciudad se genera cierta convergencia de comportamientos^6^.

Por su parte, desde un punto de vista normativo, en Colombia el espacio público es entendido como:

El conjunto de inmuebles públicos y los elementos arquitectónicos y naturales de los inmuebles privados, destinados por su naturaleza, por su uso o afectación, a la satisfacción de necesidades urbanas colectivas que trascienden, por tanto, los límites de los intereses individuales de los habitantes.^7^

La Ley 9 de 1989 señala que son diferentes las áreas que constituyen el espacio público de una ciudad, las cuales tienen diversos usos: para la circulación peatonal y vehicular; para la recreación pública, activa o pasiva; para la seguridad y tranquilidad de los ciudadanos; para la instalación y mantenimiento de los servicios públicos básicos; para el montaje y uso de los elementos que hacen parte del amoblamiento urbano; para la conservación de las obras de interés público y de los elementos históricos, culturales, religiosos, recreativos y artísticos; para el cuidado del paisaje y los elementos naturales del entorno de la ciudad. En términos generales, se trata de todas las zonas en las que se manifiesta el interés colectivo y que se constituyen para el uso y el disfrute común^7^.

El espacio público es producto de las prácticas socioculturales y políticas; en él se manifiesta la cohesión social y la lucha^2^. Pensar el espacio público como espacio de construcción de ciudadanía, se fundamenta en la reflexión sobre lo público y lo privado, así como sobre los problemas de la accesibilidad, la transparencia y la libertad; pero también implica aspectos relacionados con la promoción y el control de la sociabilidad y el encuentro social; en este sentido, hay una doble dimensionalidad del espacio público: política y urbana^8^.

Se ha señalado que las transacciones entre las personas y sus ambientes físicos tienen un rol relevante en las posibilidades y dinámicas de las interacciones sociales, como también en la realización de cierto tipo de actividades; así, la influencia del ambiente incluye el acceso a la interacción^4^^,^^9^; en este sentido, se hace referencia a otra dimensión del espacio público: la social. Desde la perspectiva de Gehl^10^, un espacio público es de calidad cuando en él, por sus características, tienen lugar no solo las actividades estrictamente necesarias en la cotidianidad de la vida de los ciudadanos, sino también, muchas actividades no indispensables, opcionales; esto es, cuando las personas salen a los espacios públicos como un objetivo en sí mismo, para el disfrute de estos y de las posibilidades que otorgan; igualmente, cuando como resultado de actividades necesarias y opcionales hay lugar para el encuentro, para ver y oír a otras personas, para el contacto humano con diversos grados de intensidad. No obstante, desde las denominadas “tesis terminales”^11^, asistimos a una gradual pérdida de los espacios públicos como lugares de encuentro. En las sociedades actuales, el espacio público, de libre acceso y tenencia común, si es que existiera, se pone en entredicho^8^^,^^9^. 

### Los vínculos con los lugares y la amenaza de su posible disrupción

Desde el punto de vista de Moser^4^, la relación de las personas con el espacio tiene particularidades asociadas a la especificidad espacial de su configuración, al tipo de lazos sociales y actividades humanas con asiento en él, y a las oportunidades efectivas de transformación y control que tienen las personas respecto de este. En este marco, y considerando que -como se ha señalado- las distintas escalas espaciales son susceptibles de reconfiguración a partir de condiciones y circunstancias específicas, vale afirmar que la crisis sociosanitaria por covid-19 no solo implicó disrupciones en las disposiciones y relaciones con los lugares privados, íntimos y más próximos, sino también con aquellos de carácter público: es el caso de la ciudad, de la vida urbana o “entre los edificios”, la cual, de acuerdo con Gehl^10^, se modifica con las variaciones en la situación de la sociedad.

Estas relaciones con los lugares, entendidos como espacios transformados y cargados de significados^12^^,^^13^^,^^14^, pueden caracterizarse por vínculos duraderos, importantes y experimentados de forma positiva, hacia lugares que no necesariamente tienen una delimitación del todo especificada^15^. Lazos afectivos conocidos como *apego al lugar* que, desde la perspectiva de algunos autores^16^^,^^17^, puede concebirse a partir de un marco organizativo tridimensional, que involucra a personas, procesos y lugares. La primera de estas dimensiones alude a los sujetos que se apegan a los lugares, a sus experiencias en el lugar, a partir de las cuales lo significan. La segunda hace referencia a los procesos psicológicos involucrados en este vínculo: afectivos (conexión emocional con el lugar), cognitivos (recuerdos, creencias, conocimientos, significados asociados por las personas a sus entornos) y conductuales (en tanto este apego se expresa a través de acciones en relación con el lugar); es decir, apunta a la naturaleza de las interacciones psicológicas que ocurren en el lugar. La tercera dimensión, por su parte, está asociada a las características del lugar de apego, bien sean físicas o sociales^16^^,^^17^. Estas dimensiones, para algunos autores, pueden ser comprendidas y abordadas de forma independiente, mientras que, para otros, son indiscernibles separadamente.

Ahora bien, el apego a los lugares puede verse amenazado por diversas situaciones. Consecuentemente, se ha señalado que, ante la potencial destrucción o necesidad de abandono forzoso del lugar, el sufrimiento asociado a esta pérdida pone de manifiesto la intensidad de tal vínculo, así como los efectos de su posible disrupción. Para Brown y Perkins^15^, toda transformación *en* el lugar o *del* lugar de apego representa una disrupción, que puede derivar en consecuencias psicosociales dependientes de los antecedentes del vínculo. Cabe señalar que, para estos autores, el cambio es inherente a las personas, los procesos psicológicos y los lugares, por lo que las dimensiones del apego al lugar son dinámicas, están en movimiento permanente. 

En este sentido, resulta relevante analizar aspectos de las relaciones con los lugares en el marco disruptivo de la pandemia por covid-19, que derivó en cambios *en* y *de* los espacios, en confinamientos y restricciones al tránsito, a la movilidad y a la permanencia en ciertos espacios, en una mayor regulación de los usos espaciales y de las interacciones sociales en el ámbito colombiano como en otros contextos; asunto que implicó nuevas relacionalidades con las espacialidades cotidianas, no solo las privadas y más próximas, sino también en otros niveles de referencia espacial, como en los ambientes públicos^4^. Esto último puso de manifiesto las profundas contradicciones, inequidades, disputas del espacio público urbano, ya antes problematizadas por muchos autores. 

Con el fin de hacer una aproximación a estas cuestiones, reflexionamos en torno a la relación de las personas con el espacio público, en el marco de la crisis sociosanitaria por covid-19. El artículo se propone, entonces, presentar una reflexión teórica a partir del estudio de la bibliografía de interés, lo cual nos permitió pensar las restricciones pandémicas y los usos y relaciones con el espacio público en el caso de Colombia. Hemos desarrollado esta reflexión teórica alrededor de cuatro categorías que se tornaron ejes de análisis relevantes: las transformaciones de la espacialidad pública en tanto materialidad; los usos normativos del espacio público como forma de vigilancia y control de los cuerpos; la reducción del mundo a escalas proximales del lugar y las consecuentes transformaciones en las relacionalidades *con* y *en* el espacio público; y, las contradicciones e inequidades del espacio público, expresadas en la distribución desigual de las formas espaciales de vivir la pandemia. Esta reflexión está enmarcada en el proyecto de investigación “Disrupciones y reconfiguraciones socioespaciales durante la pandemia por covid-19 en la comunidad universitaria, Universidad de Antioquia”, el cual cuenta con el aval del Comité de Ética del Área de Ciencias Sociales, Humanidades y Artes de la misma institución.

## RELACIONES CON EL ESPACIO PÚBLICO Y PANDEMIA POR COVID-19

### Las transformaciones de la espacialidad pública como materialidad

La crisis por covid-19 implicó en Colombia, como en otras geografías, transformaciones en la espacialidad material, derivadas de una necesaria reconfiguración de ciertas disposiciones de infraestructura, en función de hacer frente a las demandas de bioseguridad incorporadas con la situación sanitaria; desde el acondicionamiento de dispositivos para la limpieza de manos, la ubicación de marcaciones que informaban en qué lugares debían situarse las personas, y el ordenamiento de los espacios para la realización de actividades diversas (que determinaba dónde sentarse, pararse, comer, hacer deporte, etc.), hasta cierres parciales o totales de algunos espacios, en los que no pudo tener lugar la actividad exterior e implicó la pérdida de la calle como espacio jugable, para la realización de actividades necesarias, opcionales y sociales^10^^,^^18^^,^^19^, pérdida que enfatizó el principio segregacionista del espacio urbano biopolítico, ya analizado por autores como Castro Orellana^20^.

Es relevante destacar que estas transformaciones en las materialidades de los espacios públicos no estuvieron necesariamente planificadas. Esto, sumado a la desigualdad en el acceso a estas espacialidades, generó, de acuerdo con Padilla^21^, afectaciones diferenciales en el bienestar de ciertos grupos poblacionales, en la medida en que limitó los espacios de recreación, de interacción con la naturaleza y con otras personas. Así, grupos como los de la población mayor, vivieron una profunda experiencia de confinamiento.

Las transformaciones físicas del espacio tienen correlatos en la dimensión simbólica de la relación de las personas con los lugares, de acuerdo con una materialidad del espacio. Así, la pregunta por las relacionalidades personas-espacio involucra tanto la materialidad del espacio, sus transformaciones y usos, como los cambios en las memorias y experiencias de los sujetos *en* y *con* estas espacialidades.

### Los usos normativos del espacio público como forma de vigilancia y control de los cuerpos

Otra ruta de reflexión es justamente la asociada con los usos normativos del espacio y el gobierno de los cuerpos en el espacio público, por la vía de la vigilancia y del control social. Esta espacialidad se sujeta a “mecánicas de control y regulación” reforzadas durante la pandemia, aunque no exclusivas de esta^22^. Tanto en esta situación crítica como en otras, asociadas por ejemplo a la movilización social, el orden del espacio público es restituido por la vía de la excepcionalidad, la cual siempre implica un determinado orden de clase, género, racial, sexual; por tanto se expresa a través de una cierta “biopolítica del orden público”, que implica una delimitada distribución de los cuerpos en el espacio: quién, cómo, bajo qué condiciones-limitaciones puede transitar, por cuál espacio público. A esto se suma una militarización de la gestión sanitaria como compensación material y simbólica de las deficiencias sistemáticas en la respuesta en salud^23^^,^^24^. 

Desde esta lógica, siguiendo a Vásquez y Tapia^25^, se ha apelado a la sanitización social, comprendida como una utilización estratégica de la norma, mediada por el interés de dejar al margen a los “indeseables del espacio público” (como los vendedores ambulantes, para el caso de su estudio), usando la pandemia como argumento para “ordenar, desplazar e higienizar calles y aceras”.

Las regulaciones pandémicas estuvieron dirigidas a un minucioso control y registro de las interacciones de los cuerpos en los espacios. Así, los Estados emprendieron todo un proceso de biocontrol desde afuera, con protocolos científicamente aprobados que fueron

…decantando “adaptaciones sanitarias” sobre acciones tan mundanas como saludarse, dialogar, estornudar, tocarse la nariz o la boca. Tanto a nivel “macro” como a nivel “micro”, la vida en la pandemia va moldeando un cuerpo marcado por un evento epidemiológico omnipresente.^26^

Este moldeamiento de los cuerpos adquirió un lugar en el espacio público donde se implementaron las restricciones, pues allí se dan interacciones corporales y espaciales que requieren un control interno y externo; en la medida en que el cuerpo representa un riesgo, una amenaza biológica, hay que controlarlo. Esto promueve el aislamiento, el individualismo, la distancia cada vez más marcada con los otros, lo que permite preguntarse si la pandemia potencializó la idea de un sujeto solo, encerrado en sí mismo, neoliberal. 

### Reducción del mundo a escalas proximales del lugar: Transformaciones en las relacionalidades con y en el espacio público

La situación sociosanitaria por covid-19 produjo transformaciones en la realidad espacio-temporal de las ciudades, y afectaciones de la dimensión urbana y de la relación persona-ambiente en distintas escalas. A nivel internacional, las medidas para hacer frente a la pandemia implicaron la limitación del desplazamiento entre países, así como el cierre de fronteras; en términos nacionales, la restricción del tránsito entre ciudades, comunal, barrial; a escala metropolitana y urbana, la distorsión de flujos de movilidad y la reducción del radio de acción de los ciudadanos, con sus consecuentes barreras para el acceso a servicios básicos; a nivel barrial, la alteración del uso y ocupación de espacios públicos y del relacionamiento social^27^. Al respecto, Schroeder y Vilo^28^ señalan que el desplazamiento de las personas en el marco del aislamiento obligatorio por covid-19 significó un radio de influencia más reducido desde la residencia, contexto en el cual tomó fuerza el tránsito barrial, lo que significó cambios en el relacionamiento con el barrio, con los vecinos, y una percepción distinta del entorno. 

Consecuentemente, las medidas sanitarias implicaron impactos en los estilos de vida e interferencias en las relaciones sociales y con los espacios, sea por las restricciones -distanciamiento, “confinamiento no penal”^29^- o por la percepción de las personas sobre las espacialidades frecuentadas o dejadas de frecuentar. Así, espacios exteriores antes identificados como de ocio, descanso, restauración, sensación de bienestar, calidad de vida y pertenencia, pasaron a ser identificados con una sensación de miedo e inseguridad, y se transformaron los hábitos en relación con los espacios públicos, en cuestiones políticas, jurídicas, diferencias socioeconómicas y sociodemográficas. Hubo una transformación en la percepción ambiental, un tránsito de espacios valorados, posibilitadores de la actividad cotidiana en el exterior, del cumplimiento de objetivos vitales, el encuentro, el esparcimiento, a espacios temidos, peligrosos, contenedores de riesgos: la ciudad fue vista como receptora de enfermedad^18^^,^^30^. 

Las medidas implementadas con la crisis afectaron el flujo, la aglomeración y la posibilidad de movilidad, características esenciales de los espacios públicos, lo que llevó a que algunos de estos adquirieran un nuevo cariz acompañado de temor frente a la amenaza de posibles contagios^25^. Asimismo, el derecho de acceso a los espacios públicos y semipúblicos se alteró, en la medida en que hubo mayor dificultad para el uso efectivo de puestos de salud, hospitales, cementerios; una imposibilidad o alteración del relacionamiento con estos lugares. Cabe señalar que el sentimiento de pertenencia, las memorias personales y sociales, las experiencias estéticas y signos del espacio público, característicos de la cultura en los ambientes urbanos y parte de la relación persona-ambiente, se ven modificados en situaciones de calamidad pública; en este sentido, las medidas de “distanciamiento social” y con objetos, aunque efectivas para el control pandémico, al reforzar sentimientos de inseguridad y miedo, pueden tener relevantes consecuencias para la salud^18^.

Así, la crisis sociosanitaria implicó un investimento del espacio público como lugar-amenaza, bien por las características del lugar mismo, como por las relaciones sociales con asiento en él, en la medida en que el cuerpo del otro, espacialmente cercano, fue significado también como potencialmente peligroso. El espacio público, entonces, fue concebido como lugar del contagio, abierto al riesgo, un espacio contenedor de amenazas^31^. 

### Contradicciones e inequidades del espacio público: La distribución desigual de las formas espaciales de vivir la pandemia

Las restricciones pandémicas implicaron, para un sector de la población, la realización de sus actividades vitales en el contexto de la casa; no obstante, otras personas se vieron obligadas al abandono de la seguridad del hogar en función de la supervivencia, quienes tuvieron una diferenciada vivencia del lugar. En estos términos, la pandemia por covid-19 agregó una forma de inequidad en la sociedad urbana, ya desigual, en esa experiencia espacio-temporal de la ciudad y del espacio público.

Precisamente, es la distribución diferencial de los bienes y servicios urbanos el tema que ocupa la atención de varios autores a propósito de la relación entre covid-19 y espacio público. Desde esta perspectiva, es posible hablar de los *excluidos del urbanismo* para hacer referencia a una urbanización que ha dejado de lado a millones de personas, al margen de sociedades, políticas, economías, y de servicios de educación, salud y medidas preventivas, mientras que, por sus condiciones de vida, tienen mayor exposición a los riesgos en salud. En este contexto, la pandemia agudizó las contradicciones entre la urbanización globalizada y las posibilidades de una sociedad más justa y sostenible, visibilizando, además, las situaciones precarias de los grupos sociales más vulnerables. Así, las formas de vivir y sentir la pandemia estuvieron diferencialmente distribuidas entre personas más o menos favorecidas en términos socioeconómicos^18^^,^^19^^,^^27^^,^^28^^,^^30^^,^^32^ ya que, para algunos, sus entornos no facilitaron, por la ausencia de servicios o por las condiciones de densidad poblacional y hacinamiento, la implementación de medidas de bioseguridad, lo que contribuyó a un mayor riesgo en salud^32^.

En este sentido, es imposible pensar la salud y el bienestar general con inequidad, desigualdad, exclusión y sin atender a los grupos más vulnerables^30^. Frente a esto emerge la necesidad de “reequilibrar y democratizar la experiencia urbana espacio-temporal para toda la ciudadanía”^27^ y se piensa la importancia de la participación ciudadana para la toma de decisiones sobre el espacio público y para fortalecer la inclusión social, la formulación, implementación y veeduría de políticas públicas^28^^,^^32^. 

Por otro lado, las medidas sociosanitarias implementadas obligaron a un reforzamiento de la dimensión virtual del espacio, a través de actividades de educación a distancia o de empleo remoto; en este marco, se ha enfatizado la doble dimensionalidad, material y virtual, del espacio público. El ciberespacio y las redes sociales se han tornado espacio público, de información, desinformación, enseñanza-aprendizaje y encuentro social^18^. Sin embargo, cobra relevancia el interrogante planteado por Schroeder y Vilo^28^, acerca de si este espacio público virtual reproduce las desigualdades del espacio material. Entendiendo esta dimensión virtual como atada al acceso real y efectivo a bienes y servicios públicos, la respuesta es afirmativa, por lo cual toma fuerza el desafío de garantizar oportunidades de acceso a recursos tecnológicos, información, acompañamiento en el uso de estos recursos, además de mejores condiciones en el hábitat de todos los ciudadanos, toda vez que, de acuerdo con los autores, es posible pensar un fortalecimiento de la sociedad civil a partir de la dimensión virtual del espacio público (como espacio intangible), como una nueva forma de intercambio sociocultural, de vinculación social, de configuración de lazos de participación, con otros códigos y significados. Un nuevo “lugar” de interacción para el establecimiento de otros canales de organización social-comunitaria. 

## CONSIDERACIONES FINALES

### El espacio público interrogado

El concepto de espacio público es polisémico; sin embargo, se ha pensado como aquel cuya titularidad es pública, cuyo acceso es libre y gratuito, y se ha adjetivado como *abierto*, en tanto de posible circulación y movilidad; como *colectivo*, en la medida en que se ha asociado a la construcción de ciudadanía, de democracia, a la sociabilidad, al encuentro con personas diferentes, a un carácter multifuncional; como *común*, en el sentido de su pertenencia a “todos”, de la posibilidad de “acceso general” de la población en “igualdad de condiciones”; como espacialidad de lo *cotidiano*, lo que alude a que en este se expresan las prácticas culturales de las poblaciones, al construirse a partir de la actividad rutinaria de las personas, quienes parcialmente proyectan en él su vida doméstica, con asiento en esta espacialidad se relacionan con otros, construyen lazos sociales y comparten necesidades, problemáticas y reivindicaciones^33^.

Así, el espacio público es más que un fenómeno urbanístico. Responde a múltiples dominios, en tanto que alude no solo a un ámbito físico o a una materialidad que, para el caso de análisis, fue objeto de transformaciones con las medidas pandémicas, sino también a otros ámbitos interrelacionados. Es a la vez físico, relacional, político y conflictual. Es relacional en el sentido en que en él se expresan la sociabilidad y la ciudadanía, concurren diversos grupos y actividades; en él los sujetos se encuentran con otros y hacen vida urbana; es el lugar de la “accesibilidad” y la “libertad”^8^^,^^10^^,^^33^. Considerando lo planteado, el confinamiento pandémico se constituyó, entonces, en una limitación de este aspecto relacional de la espacialidad pública. 

El carácter político, por su parte, está asociado con aspectos normativos del espacio público que demarcan sus usos y apropiación, toda vez que hay formas regulatorias (explícitas e implícitas) que lo condicionan^33^; también es político dada “la naturaleza contestada del espacio público como ámbito de territorialización de procesos y luchas sociales más amplios”^11^, esto es, como espacialidad en tensión, para la reafirmación de identidades de distintos grupos sociales, para la preservación de las diferencias entre estos mediante usos diversos y en pugna del espacio público. 

Este aspecto está relacionado con el carácter conflictual del espacio público, en tanto que en este se expresa la cohesión social y también la fractura, el desgarramiento y la lucha; es, por tanto, producto de las prácticas sociales, políticas y culturales y, a la vez, incide en ellas. Si algo ha distinguido al espacio público es precisamente su heterogeneidad constitutiva, tanto de actores sociales de variado perfil -de clase, género, etario, ideológico, etc.- como de demandas, expectativas y proyectos urbanísticos y societales. Estas coordenadas teóricas se acercan a los tres elementos definitorios que Massey^34^ ha planteado respecto del espacio en sus distintas escalas, y que manifiestan su carácter procesual: 1) el espacio es producto de las relaciones sociales; 2) es la esfera de posibilidad de la heterogeneidad, el plano en donde emergen y coexisten diferentes sujetos, voces y trayectorias; consecuentemente, no hay espacio sin multiplicidad, así como tampoco hay pluralidad sin espacio; y 3) al ser producto de la relacionalidad social, el espacio es siempre devenir, es abierto e inacabado. 

La ciudad permanece en continuo movimiento, en proceso de construcción constante, como ámbito en el que se generan negociaciones cotidianas que logran determinados grados de consistencia y momentos de estabilidad y larga duración, identificables, y otros de confrontación, inestabilidad, indefinición y caos^35^. Al ser campo de tensiones, el espacio público ha sido producido bajo ciertos patrones, por ejemplo, uno heteronormativo, donde las relaciones que no responden a este son concebidas como anómalas y han de ser proscritas o silenciadas^33^. En este sentido, el espacio público se fundamenta en formas de exclusión social; consecuentemente, su carácter estrictamente público nunca ha sido pleno, ya que responde a un principio segregacionista. Así entendido el espacio público, se pone en entredicho su pertenencia a “todos” y el “acceso general” a este en “igualdad de condiciones”, y las luchas por la inclusión por parte de los sectores poblacionales marginados se tornan en una condición estructural de esta espacialidad^11^^,^^20^. 

En el contexto de la crisis sociosanitaria por covid-19, además, la gestión y el ordenamiento de los espacios públicos implicó una determinada distribución de los cuerpos, una forma de control en la que el carácter estrictamente público de esta espacialidad también estuvo en cuestión, ya no solo por su naturaleza segregacionista. Las restricciones a la movilidad y al acceso, suponen una disrupción de las cualidades del espacio público como abierto, para el flujo, la aglomeración y para la “libertad” de desplazamiento. Se trató de condicionamientos normativos de registro y control de los cuerpos en los espacios, que devinieron en control de la sociabilidad y de la apropiación de la calle como emplazamiento del encuentro y de la disputa con otros diversos, así como de la lucha social, aspectos fundamentales del espacio público; mientras que, como se señaló antes, se apeló a la sanitización social como estrategia para dejar al margen a “indeseables del espacio público”, para desplazar, reordenar e higienizar las calles^25^^,^^26^.

### La dimensión simbólica del espacio público

Por otro lado, a estas reflexiones sumaríamos el ámbito de lo simbólico respecto del espacio público, en el sentido de la construcción de lugar. El espacio público es objeto de significación, cobra sentidos particulares para las personas que lo habitan y transitan, quienes en su experiencia pueden hacer de él un lugar. A través de la interacción personas-espacios estos últimos son transformados e interiorizados mediante procesos socioafectivos y cognitivos, condicionados por las pertenencias sociales de las personas. A estas espacialidades los sujetos les otorgan sentidos, sobre ellas configuran memorias, con ellas establecen vínculos; los espacios, además, pueden ser resemantizados en el tiempo, tienen una connotación móvil, dinámica^12^. Consecuentemente, las restricciones en el tránsito y los usos de las espacialidades públicas, en el marco de la crisis sociosanitaria por covid-19 como de otros confinamientos, por ejemplo, asociados con el orden público, frecuentes en Colombia, pueden representar movilidad de sentidos de estos espacios-lugares públicos; muestra de ello es el tránsito de espacios valorados, posibilitadores de la actividad cotidiana en el exterior, del cumplimiento de objetivos vitales, el encuentro y la lucha, a espacios temidos, peligrosos, contenedores de riesgos^18^^,^^30^, lo cual remite a la necesidad de pensar el carácter simbólico de la relación de las personas con el espacio público.

Esto último, aunado a aspectos como las condiciones de inequidad, las restricciones, las transformaciones, el vaciamiento o la promoción de actividades en los espacios públicos, y el control poblacional por la vía de su normalización, nos llevan a considerar la importancia, para la comprensión de la relación de las personas con el espacio público, de la realización de acercamientos a sus experiencias en estas espacialidades, teniendo en cuenta, además, la propuesta de Schroeder y Vilo^28^ de rescatar el sentido vivencial de los espacios públicos, a partir del análisis de nuevas relaciones y redes sociales que allí tienen lugar. Vale la pena, así, volver la mirada a la dimensión simbólica de la relación personas-espacio público.
